# Melatonin regulates cancer migration and stemness and enhances the anti‐tumour effect of cisplatin

**DOI:** 10.1111/jcmm.17809

**Published:** 2023-06-12

**Authors:** Linglin Cheng, Shubo Li, Kailun He, Ye Kang, Tianye Li, Chunting Li, Yi Zhang, Wanlu Zhang, Yongye Huang

**Affiliations:** ^1^ College of Life and Health Sciences Northeastern University Shenyang China; ^2^ Liaoning Center for Animal Disease Control and Prevention Liaoning Agricultural Development Service Center Shenyang China; ^3^ Department of Pathology Shengjing Hospital of China Medical University Shenyang China; ^4^ Key Laboratory of Bioresource Research and Development of Liaoning Province, College of Life and Health Sciences Northeastern University Shenyang China

**Keywords:** cancer stem cells, cisplatin, combinatorial treatment, gastric cancer, melatonin

## Abstract

Melatonin, a lipophilic hormone released from the pineal gland, has oncostatic effects on various types of cancers. However, its cancer treatment potential needs to be improved by deciphering its corresponding mechanisms of action and optimising therapeutic strategy. In the present study, melatonin inhibited gastric cancer cell migration and soft agar colony formation. Magnetic‐activated cell sorting was applied to isolate CD133^+^ cancer stem cells. Gene expression analysis showed that melatonin lowered the upregulation of LC3‐II expression in CD133^+^ cells compared to CD133^−^ cells. Several long non‐coding RNAs and many components in the canonical Wnt signalling pathway were altered in melatonin‐treated cells. In addition, knockdown of long non‐coding RNA H19 enhanced the expression of pro‐apoptotic genes, Bax and Bak, induced by melatonin treatment. Combinatorial treatment with melatonin and cisplatin was investigated to improve the applicability of melatonin as an anticancer therapy. Combinatorial treatment increased the apoptosis rate and induced G0/G1 cell cycle arrest. Melatonin can regulate migration and stemness in gastric cancer cells by modifying many signalling pathways. Combinatorial treatment with melatonin and cisplatin has the potential to improve the therapeutic efficacy of both.

## 1 INTRODUCTION

Cancer cells can spread directly from the primary lesion to distal sites, or metastasize, by invading the lymphatic system or the bloodstream, thereby forming metastatic cancer. Metastasis makes surgical and locoregional therapy for cancer extremely difficult. In addition, metastasis is often associated with cancer stem cells identified with high global and confidence biomarker scores,^1^, ^2^ and cancer stem cells are considered as the leading factor of chemotherapeutic resistance.^2^ Therefore, the development of advanced anticancer drugs and treatment strategies is necessary to inhibit cancer cell migration.

Some chemicals secreted by the body may exhibit excellent cancer‐inhibiting effects. Melatonin, whose secretion is controlled by circadian rhythms, is a hormone synthesized and secreted mainly by the pineal gland.^3^ Many studies have shown that melatonin can effectively inhibit all stages of cancer development and enhance the sensitivity of cancer cells to various anticancer drugs.^4^, ^5^, ^6^ Melatonin is a pleiotropic anticancer molecule that regulates malignant cells via multiple mechanisms, including enhancement of mitochondrial reactive oxygen species,^7^ targeting of Wnt/beta‐catenin signalling,^8^ regulation of histone modification,^9^, ^10^ etc. In our previous study, melatonin was shown to suppress gastric cancer by modulating endoplasmic reticulum (ER) stress, autophagy and the Ras–Raf–ERK signalling pathway.^11^ However, a greater effort is needed to improve the utility of melatonin as an anticancer treatment. It is especially important to further uncover the role of melatonin in metastasis and discover suitable strategies to promote its application in cancer treatment by minimising the potential therapeutic risks.

Various studies have suggested that single‐drug chemotherapy could cause cancer cells to develop resistance, thus greatly reducing the effectiveness of cancer treatment. Combination therapies could reduce the likelihood of acquired resistance, shorten the duration of treatment and speed up recovery.^12^, ^13^ Cisplatin, a platinum‐containing anticancer drug, is a non‐specific modulator of the cell cycle and exhibits a strong anticancer effect by interacting with N^7^‐guanine and N^3^‐adenine on cancer cells, damaging the structures on the cell membrane, and inhibiting replication of cancer cells, ultimately leading to cancer cell necroptosis.^14^, ^15^, ^16^, ^17^ However, cisplatin also causes severe toxicities, including nephrotoxicity, peripheral neuropathy and ototoxicity. Long‐term exposure to cisplatin could cause cancer cells to develop drug resistance.^16^, ^18^ We conducted an in‐depth study of the anticancer effects of melatonin and cisplatin combination therapy with a view to identifying new diagnostic markers and developing appropriate therapeutic approaches for cancer patients, as well as to broadening the application of both melatonin and cisplatin in cancer therapy.

## 2 MATERIALS AND METHODS

### 2.1 Cell culture

Human gastric cancer cells, SGC‐7901, were cultured in DMEM medium supplemented with 10% fetal bovine serum, 1% non‐essential amino acids, 1% glutamine, 100 U/mL penicillin and 100 mg/mL streptomycin at 37°C in 5% CO_2_ atmosphere. Melatonin (Sigma‐Aldrich) was prepared in dimethyl sulfoxide (DMSO) at 5.0 mol/L and diluted with medium to create different concentrations.

### 2.2 Flow cytometry analysis of apoptosis

After incubation with melatonin (2.5 mmol/L) and/or cisplatin (0.25, 0.5, 2.5 or 5.0 mmol/L) for 24 h, cells were digested with 0.25% trypsin, collected after centrifugation by gentle blowing and washed twice with cold phosphate buffer. The Annexin V‐FITC/PI apoptosis detection kit was then applied. In brief, 100 μL of 1× binding buffer was added to dilute the precipitate, followed by addition of 5 μL of Annexin V‐FITC and 5 μL of PI. After incubation at room temperature (in the dark) for 15 min, 400 μL of 1× binding buffer was added and the solution was immediately analysed using flow cytometry (Fortessa, BD Biosciences).

### 2.3 Cell cycle distribution analysis

Gastric cancer cells were treated with melatonin and/or cisplatin for 24 h, collected in 0.1% trypsin and 2.5 mmol/L EDTA, and washed using cold phosphate buffer. The cells were fixed using cold 70% ethanol and refrigerated overnight at −20°C. They were then washed with phosphate buffer, and 500 μL of PI/RNase staining buffer was added to them. The cells were incubated for 30 min at room temperature then analysed using a flow cytometer (Fortessa, BD Biosciences).

### 2.4 Real‐time quantitative PCR

Real‐time quantitative PCR (RT‐qPCR) was used to determine gene expression at transcriptional level in gastric cancer cells. RNA was isolated using TRIzol reagent, and the purity of RNA was determined according to the instructions. Briefly, 2 μg of RNA was reverse‐transcribed into complementary cDNA using the All‐in‐One cDNA Synthesis SuperMix kit (Bimake). RT‐qPCR was performed with 2× SYBR Green qPCR Master Mix (Bimake) on a CFX96 real‐time PCR detection system. Relative expression of target genes was calculated using the 2^−ΔΔCt^ method based on analysis of amplification and melting curves. All experiments were repeated three times.

### 2.5 Western blotting

After being washed with PBS twice, the cells were lysed with phenylmethane sulfonyl fluoride lysis buffer. Total protein was collected by centrifugation and concentration was measured using a bicinchoninic acid protein assay kit (Beyotime). Next, 20 μg of the extracted protein samples was separated by electrophoresis on 12% sodium dodecyl sulfate‐polyacrylamide gel and transferred to polyvinylidene difluoride membranes. The membranes were blocked with 5% skim milk and incubated at room temperature for 1 h. The primary antibody was added and incubated overnight at 4°C. After being washed with TBST three times, the membranes were incubated for 1 h with the appropriate amount of secondary antibody. Protein bands were visualized using an enhanced chemiluminescence detection system.

### 2.6 Soft agar colony formation assay

To each well of a 6‐well plate, 1.5 mL of 0.6% agar containing 10% FBS, which served as the agar base, was added and allowed to solidify. Then, 500 μL of 0.6% agar solution was mixed with 500 μL of DMEM containing 20% FBS and 3000 gastric cancer cells treated with 0, 1.0, 2.5 or 5.0 mM melatonin. These mixtures were seeded on the top of the agar base and allowed to solidify. Cells were then incubated in a 37°C, humidified, 95% air and 5% CO_2_ atmosphere to form colonies. Next, 100 μL of DMEM containing 10% FBS was added to the wells every 3 days. The colony assay was terminated at Day 21 and colonies were stained with 0.1% crystal violet solution. Colonies with a diameter larger than 50 μm were counted and visualized under a microscope.

### 2.7 Cell migration assay

The cell migration assay was performed using 24‐well‐plate transwell chambers (8 μm pore size). In brief, 2 × 10^4^ gastric cancer cells in 200 μL serum‐free DMEM were seeded into the upper chamber of the transwell insert, while 600 μL of 2.5% FBS‐containing DMEM was placed in the bottom chamber. After 24 h of incubation, cells that moved from the upper surface of the membrane to the bottom of the membrane were fixed in methanol for 30 min at room temperature and stained with 0.1% crystal violet for another 20 min. The migrated cells were then observed using an optical microscope.

### 2.8 Isolation of CD133 ^+^ cancer stem cells with magnetic‐activated cell sorting system

Treated gastric cancer cells were harvested, and CD133^+^ cells were isolated using the MACS indirect CD133 MicroBead kit (MiltenyiBiotec) according to standard enrichment procedures. In brief, cells were labelled with CD133 micro‐magnetic beads and incubated at 4°C for 30 min in the dark. CD133^+^ and CD133^−^ cells were isolated separately using the sorting column. Cell sheet precipitates were obtained by centrifugation and prepared as cell suspensions using PBS buffer.

### 2.9 Statistical analysis

Data are expressed as mean ± standard error of the mean (SEM). Statistical analyses were performed using SPSS software, and statistically significant differences were determined using one‐way analysis of variance (anova). *p* < 0.05 was considered statistically significant. Experiments were conducted in triplicate.

## 3 RESULTS

### 3.1 Melatonin‐silenced cell migration and remodelled cytoskeleton

**FIGURE 1:**
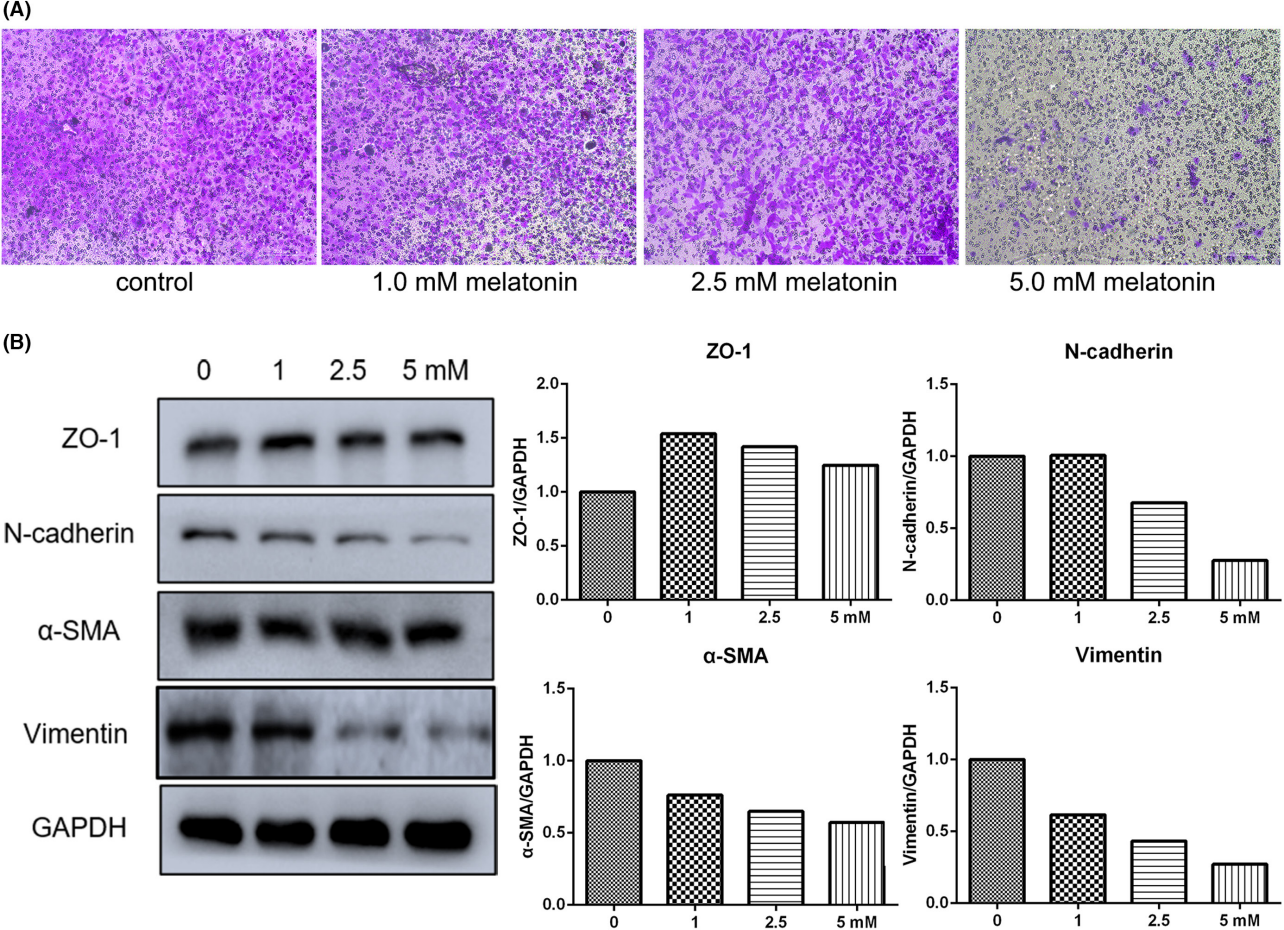
Treatment with melatonin (24 h) suppressed the migration of cancer cells. (A) Cell migration determined by transwell assay. (B) Epithelial–mesenchymal transition (EMT) gene expression evaluated by western blot.

Gastric cancer cells were treated with different concentrations of melatonin (0, 1.0, 2.5 and 5.0 mM) and evaluated using the transwell assay to investigate the effects of melatonin on their migration ability. Cell migration was reduced in the presence of melatonin (Figure 1A) and was dramatically inhibited in cells treated with 5.0 mM melatonin. Epithelial–mesenchymal transition (EMT) is considered the inceptive step of tumour invasion and metastasis. Therefore, we evaluated the expression of EMT‐related genes. Results of RT‐qPCR indicated that fibronectin expression was significantly downregulated in cells treated with 2.5 or 5.0 mM melatonin (Figure S1). In addition, Snail and Zeb1 expression significantly increased in cells treated with 2.5 or 5.0 mM melatonin. However, no significant differences were observed in the expression of E‐cadherin, N‐cadherin, Slug, Twist1 and the Zeb2 at transcription level between different groups. The results of western blotting showed that ZO‐1 expression slightly increased after melatonin treatment. The expression of N‐cadherin, vimentin and α‐SMA was downregulated at the protein level in cells treated with melatonin. Vimentin expression was reduced at the transcriptional level in cells treated with 2.5 or 5.0 mM melatonin (Figure 1B). Thus, these results suggest that melatonin is an inhibitor of gastric cancer cell migration via modulation of many EMT‐associated genes.

**FIGURE 2:**
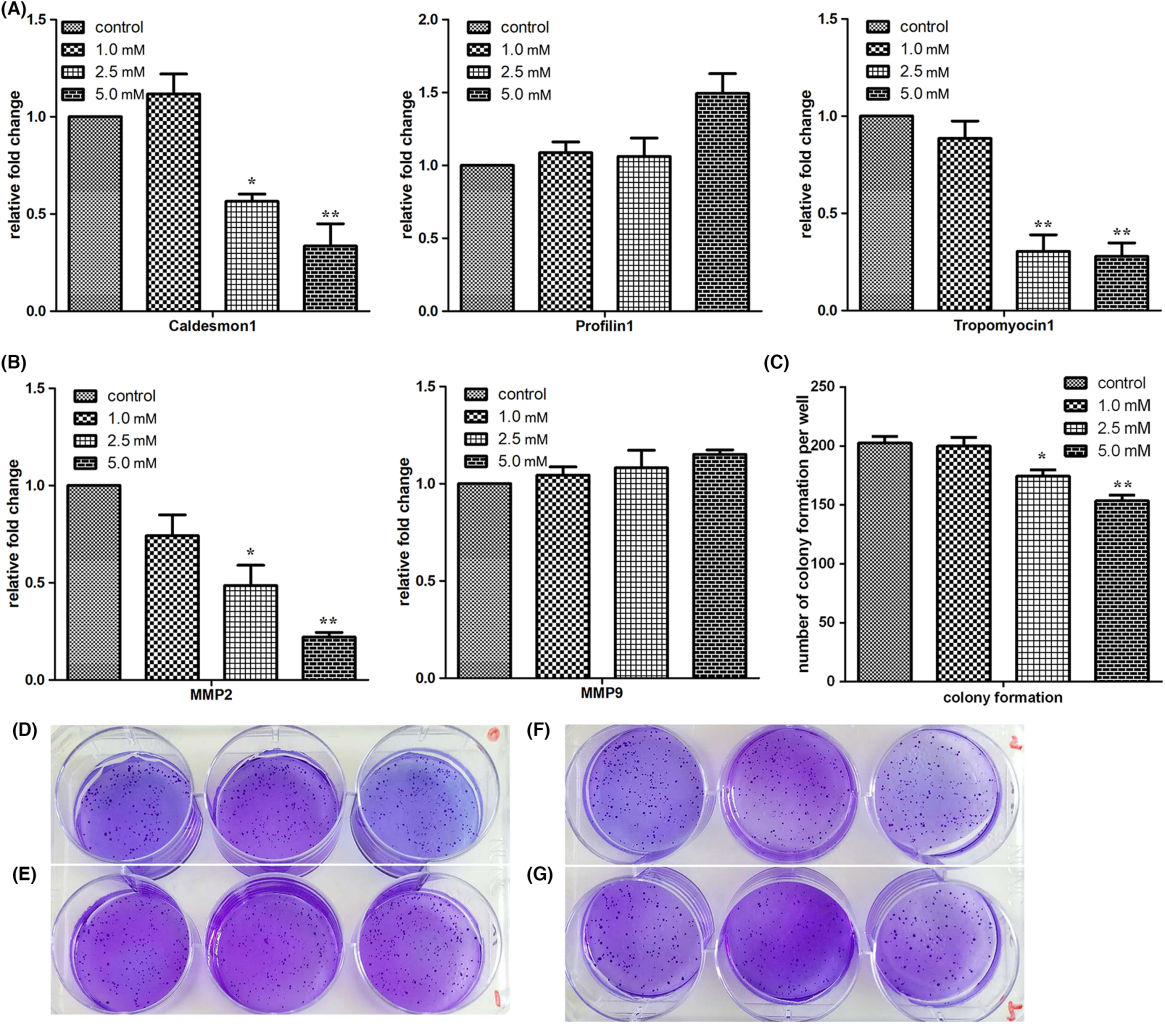
Treatment with melatonin (24 h) impaired the expression of cytoskeleton and matrix metalloprotein‐associated genes. (A) Expression of cytoskeleton‐related genes evaluated by RT‐qPCR. (B) Expression of matrix metalloprotein associated genes as determined by RT‐qPCR. (C–G) Self‐renewal capability of cells treated with 0 (D), 1.0 (E), 2.5 (F) and 5.0 mM (G) of melatonin, assessed by soft agar assay. Data are presented as mean ± SEM of duplicate experiments. *p < 0.05 and **p < 0.01, indicate significant differences relative to the control group.

As cell migration is closely related to cytoskeleton reorganisation, the expression of caldesmon 1, profilin 1 and tropomyosin 1 was analysed. Caldesmon 1 and tropomyosin 1 expression was reduced in SGC7901 cells treated with 2.5 or 5.0 mM melatonin (Figure 2A). These results suggest that the cytoskeleton can be remodelled by melatonin treatment. Within malignant tumours, matrix metalloproteinases (MMPs) presenting as inactive zymogens are mainly secreted by mesenchymal cells. Activated MMPs can mediate cellular degradation of host ECM, control tumour neovascularization, regulate cell adhesion and modulate tumour cell growth by affecting intracellular signalling. They are directly or indirectly involved in a variety of physiological and pathological processes. Both MMP2 and MMP9 are capable of regulating migration and invasion and serve as important prognostic factors of many cancers. MMP2 expression was significantly downregulated in cells exposed to 2.5 or 5.0 mM melatonin, but no significant differences were observed in MMP9 expression between control cells and melatonin‐treated cells (Figure 2B). In addition, self‐renewal capability was assessed by soft agar assay, and results showed that treatment with melatonin decreased colony formation (Figure 2C–G). In general, many indices associated with metastasis were reduced after melatonin treatment.

### 3.2 Melatonin treatment participated in the regulation of cancer stemness

**FIGURE 3:**
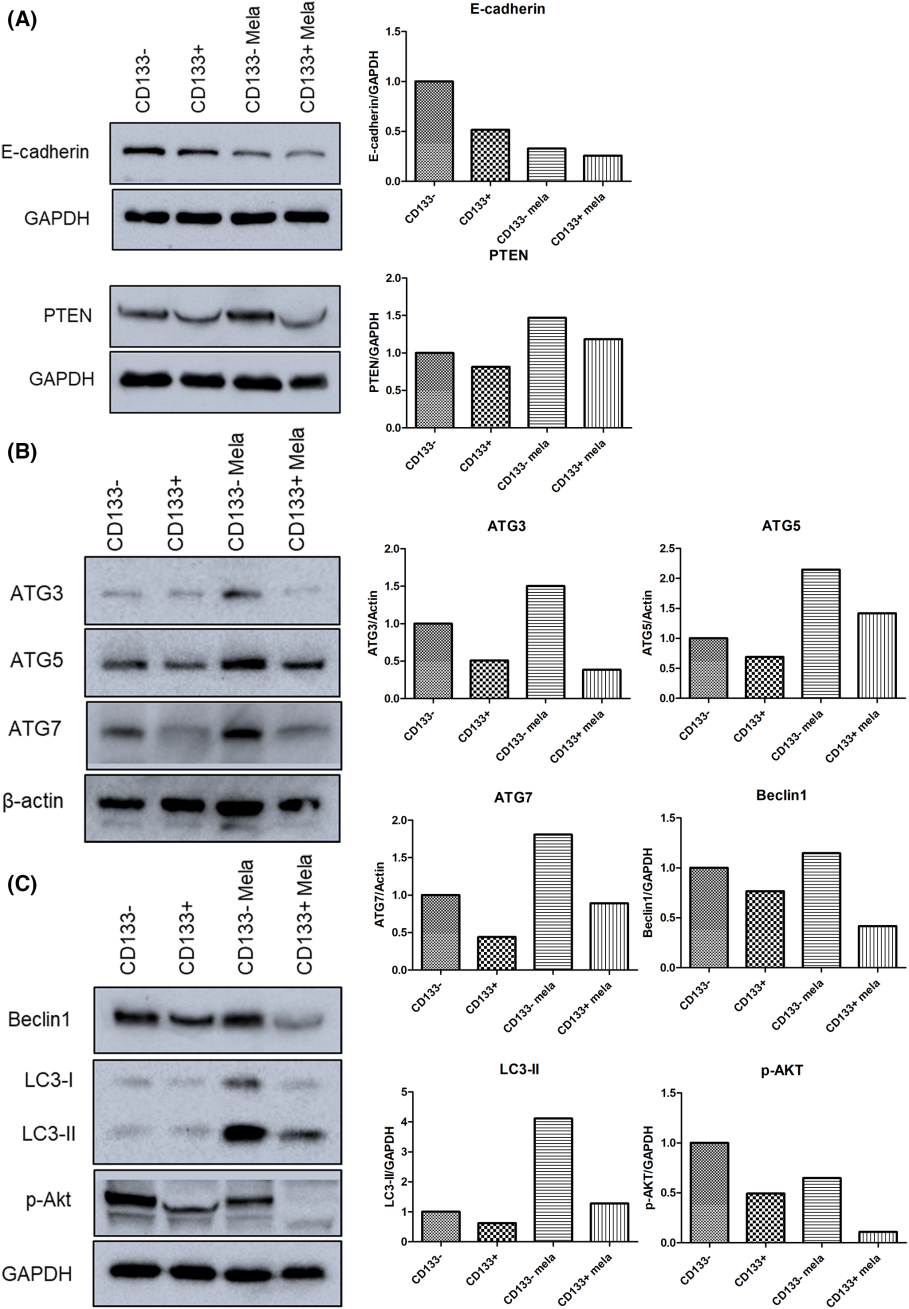
Gene expression of CD133^+^ cells treated with melatonin analysed using western blot. (A) Expression of proteins related to cell progression and invasion. (B, C) Expression of proteins associated with cellular autophagy.

Cancer stem cells show extreme tumorigenic capacity in inducing tumour proliferation, recurrence and metastasis. Considering the significant role played by cancer stem cells in cancer development, it is meaningful to explore the effects of melatonin on cancer stem cells. CD133 is a potential marker of gastric cancer stem cells. Therefore, we examined gene expression in CD133^+^ cells treated with melatonin. E‐cadherin expression in CD133^+^ cells was reduced compared to that in CD133^−^ cells (Figure 3A). In addition, after being treated with 2.5 mM melatonin, CD133^+^ cells showed further downregulation of E‐cadherin expression, indicating that cancer stem cell metastasis might be exacerbated after melatonin treatment. PTEN is a widely studied tumour suppressor. In the present study, PTEN expression in CD133^+^ cells was lower than that in CD133^−^ cells. PTEN expression could be promoted by melatonin treatment, although its expression in melatonin‐treated CD133^+^ cells was lower than in melatonin‐treated CD133^−^ cells. In general, anti‐tumour mechanisms were likely activated by melatonin.

Tumorigenesis is believed to be associated with dysregulation of cellular autophagy. Autophagy has both tumour promotion and suppression functions depending on context. Therefore, the expression of autophagy‐related genes, ATG3, ATG5, ATG7, Beclin1, LC3 and phosphorylated Akt (p‐Akt), was examined using western blotting. ATG3, ATG5, ATG7 and Beclin1 expression in CD133^−^ cells was enhanced by melatonin treatment (Figure 3). In CD133^+^ cells, ATG5 and ATG7 expression was promoted by melatonin treatment. As expected, LC3‐II expression increased in melatonin‐treated cells, but its expression in melatonin‐treated CD133^−^ cells was lower than that in melatonin‐treated CD133^−^ cells. In addition, p‐Akt expression in both melatonin‐treated CD133^+^ and CD133^−^ cells was lower than that in non‐treated cells. These results indicate that autophagy might play a regulatory role in the stemness of melatonin‐treated cancer cells.

### 3.3 Wnt signalling pathway involved in melatonin treatment

**FIGURE 4:**
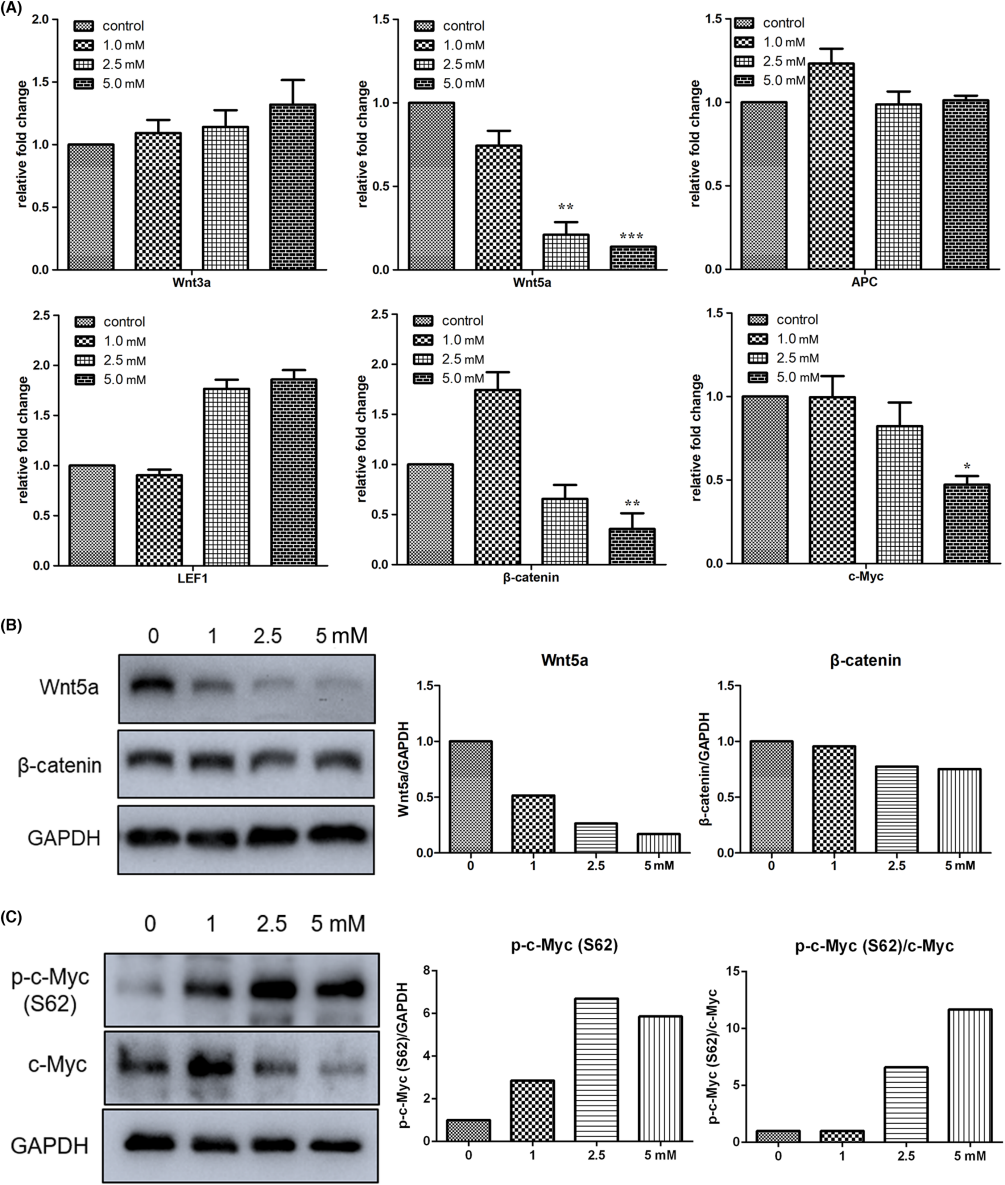
Treatment with melatonin (24 h) inhibited the expression of the Wnt signalling pathway. (A) Expression of Wnt signal components determined by RT‐qPCR. (B) Expression of Wnt5a and β‐catenin determined by western blot analysis. (C) Expression of c‐Myc evaluated by western blot analysis. Values are mean ± SEM of duplicate experiments. *p < 0.05, **p < 0.01 and ***p < 0.001, indicate significant differences relative to the control group.

Wnt/β‐catenin is a key signalling pathway in the regulation of cancer metastasis and stemness. Thus, several components (Wnt3a, Wnt5a, APC, LEF1, β‐catenin and c‐Myc) of the canonical Wnt signalling pathway were evaluated in this study. Results of RT‐qPCR showed that Wnt5a expression significantly decreased in cells treated with 2.5 or 5.0 mM melatonin; β‐catenin and c‐Myc expression was also significantly downregulated by 5.0 mM melatonin (Figure 4A). No significant differences were observed in Wnt3a, APC and LEF1 expression between the control and melatonin‐treated groups. Western blotting was performed to confirm the gene expression profile, and results showed that melatonin treatment decreased Wnt5a and β‐catenin expression in a dose‐dependent manner (Figure 4B). These results suggest that melatonin treatment may lead to inhibition of the expression of many components in the canonical Wnt signalling pathway.

Myc is a proto‐oncogene, and the phosphoprotein of c‐Myc is important for apoptosis, cell cycle progression and cellular transformation. In addition, c‐Myc is a target gene related to the Wnt signalling pathway. In the present study, c‐Myc expression in gastric cancer cells treated with 2.5 or 5.0 mM melatonin was downregulated (Figure 4C). The protein stability of c‐Myc is regulated by two phosphorylation sites: threonine 58 (T58) and serine 62 (S62). Our data showed that melatonin promoted S62 phosphorylation in c‐Myc (p‐c‐Myc (S62)) in a dose‐dependent manner. Most importantly, the ratio of p‐c‐Myc (S62)/c‐Myc increased in the cells treated with 2.5 or 5.0 mM melatonin. These data further confirm the downregulation of Wnt signalling by melatonin; however, the stability of c‐Myc was likely promoted after melatonin treatment.

### 3.4 LncRNA H19 knockdown enforced melatonin‐induced cancer cell death

**FIGURE 5:**
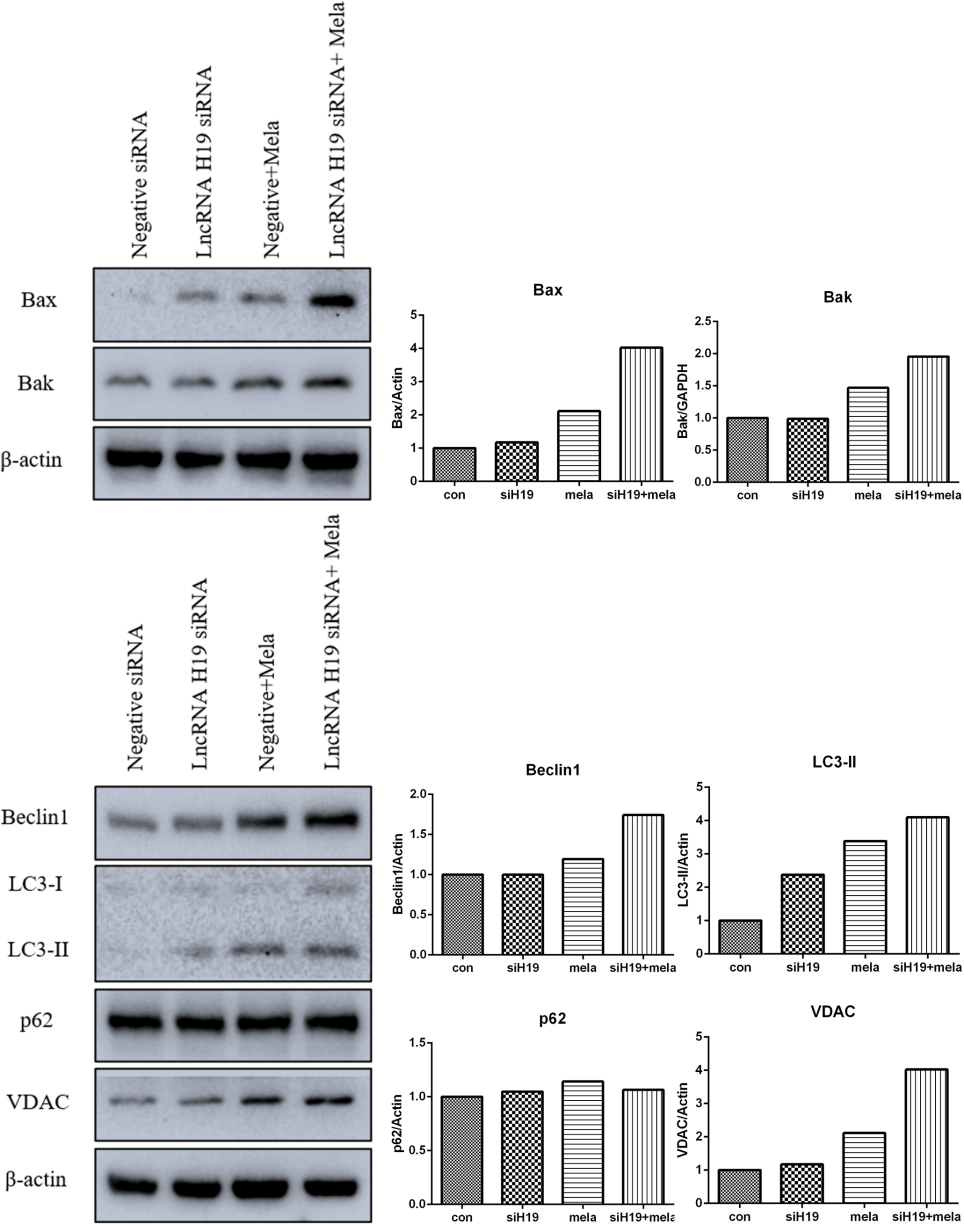
Expression of apoptosis and autophagy‐related proteins in melatonin‐treated gastric cancer cells with lncRNA H19 knockdown.

Long noncoding RNAs (lncRNAs) regulate various biological processes, including proliferation, apoptosis, metastasis and stemness. Our previous study showed that melatonin altered the expression of lncRNAs.^11^ Therefore, the present study attempted to further uncover the role of melatonin treatment in lncRNA expression. As shown in Figure S2, LINC01121 expression was upregulated in cells treated with 5.0 mM melatonin. lncRNA COX2 expression also increased in a dose‐dependent manner in cells treated with melatonin. In contrast, lncRNA H19, TUG1, HOTAIR, MALAT1, SAMMSON and UCA1 expression was inhibited in cells treated with 2.5 or 5.0 mM melatonin. Our previous study showed that knockdown of lncRNA H19 expression could enhance the anti‐tumour effect of natural products. Thus, to further determine the effects of lncRNA H19 on melatonin, siRNAs were applied to knockdown its expression and 2.5 mM melatonin was applied in combination with H19 siRNA. As shown in Figure 5, Beclin 1 and VDAC expression was also upregulated in melatonin‐treated cells, and their expression further increased after lncRNA H19 knockdown. In addition, LC3‐II expression was promoted in the melatonin‐treated cells with lncRNA H19 knockdown, indicating that autophagy was further activated. It is well known that excessively activated autophagy cascades can provoke autophagic cell death. As expected, Bax and Bak expression was increased by melatonin treatment, and their expression was further enhanced in melatonin‐treated cells with lncRNA knockdown. MLKL, a marker of necroptosis, was also overexpressed in cells receiving the combinatorial treatment of melatonin and lncRNA H19 siRNA (Figure S3). ER stress is closely linked to apoptosis and autophagy. Our results show that Bip and CHOP expression increased in melatonin‐treated cells with lncRNA H19 knockdown. The above findings indicate that knockdown of lncRNA H19 assisted melatonin in inducing cancer cell death.

### 3.5 Melatonin in combination with cisplatin enhanced the induction of cancer cell apoptosis

**FIGURE 6:**
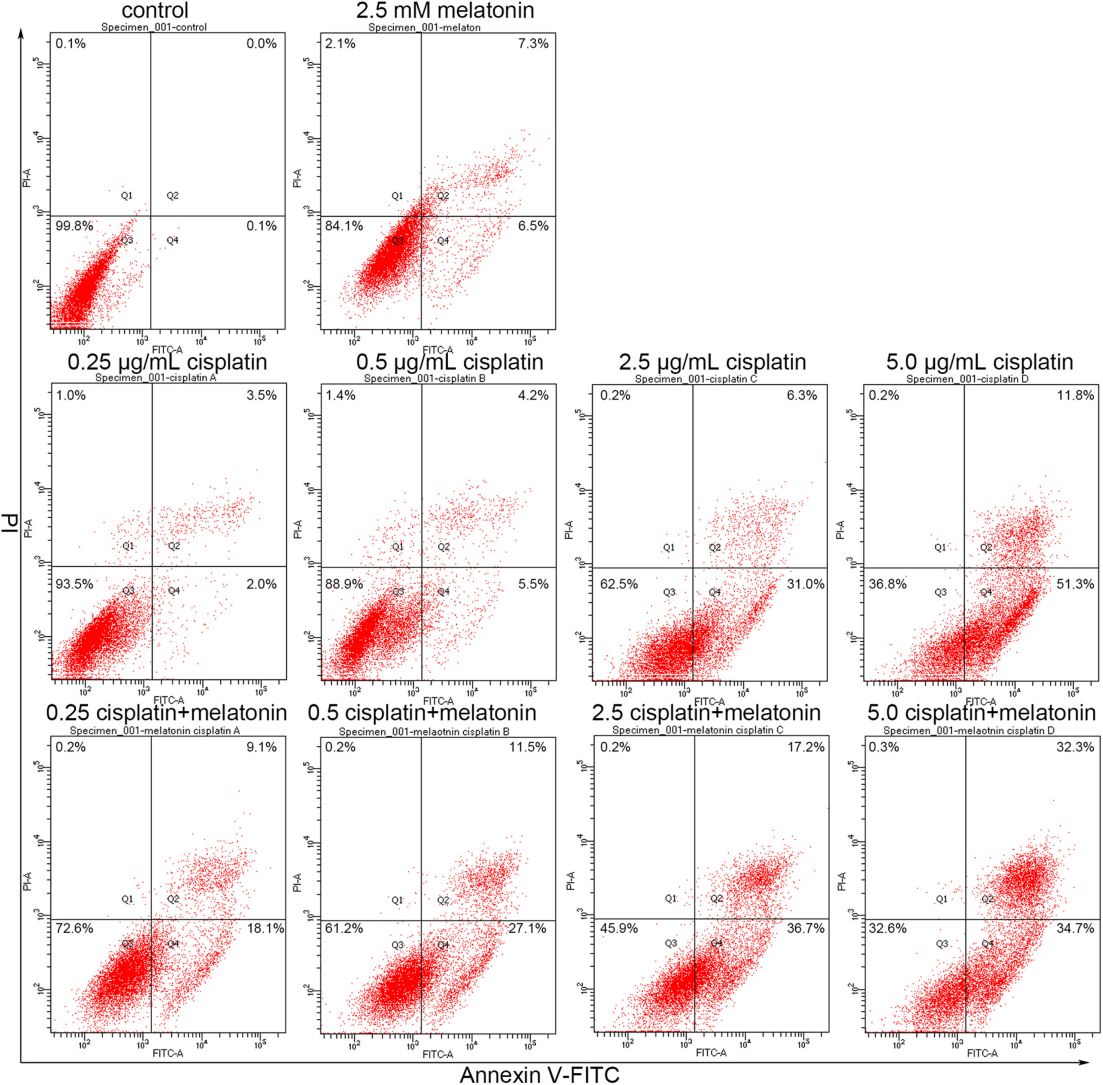
Combinatorial treatment with melatonin (2.5 mM) and cisplatin (0.25, 0.5, 2.5 or 5.0 μg/mL) for 24 h promoted apoptosis in gastric cancer cells. Apoptosis was evaluated by flow cytometry with Annexin V/PI staining.

**FIGURE 7:**
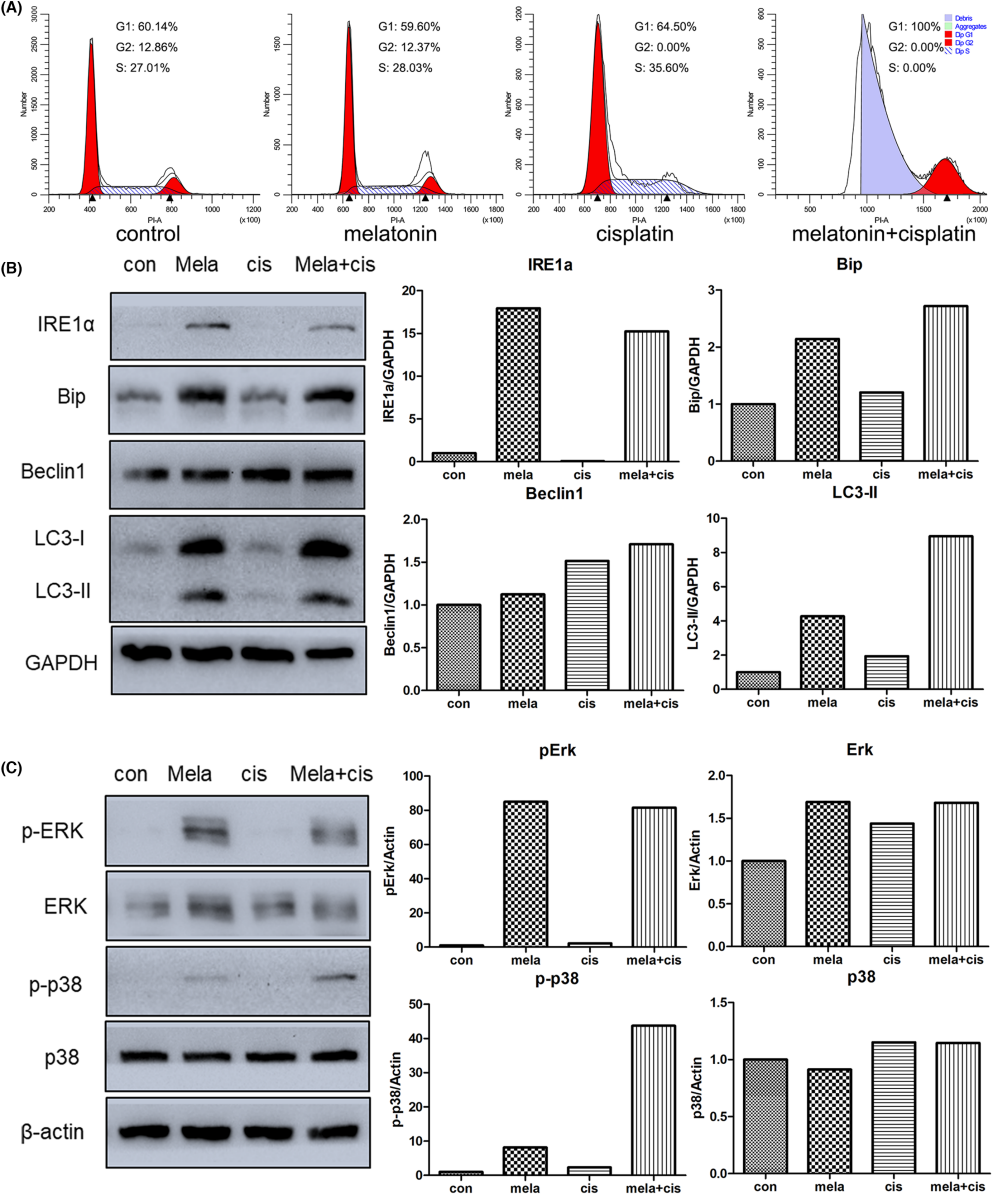
Effects on gastric cancer cells of combinatorial treatment with melatonin (mela, 2.5 mM) and cisplatin (cis, 0.5 μg/mL) for 24 h. (A) Cell cycle distribution determined by flow cytometry analyses using PI staining. (B) Expression of endoplasmic reticulum (ER) stress or autophagy‐related genes determined by western blot analysis. (C) Expression of MAPK‐associated genes evaluated by western blot analysis.

The above results suggest that melatonin treatment could be effective in cancer cell inhibition. However, a large number of residual cells still remained after melatonin treatment. Therefore, combinatorial treatment might be an appropriate strategy to strengthen the effects of melatonin on cancer cells. Cisplatin is a classic platinum‐based anticancer drug widely used to treat various types of cancers. In the present study, the therapeutic outcomes of combining melatonin with cisplatin were investigated. Apoptosis was assessed first via the Annexin V‐FITC/PI assay in cells that underwent combinatorial treatment with melatonin (2.5 mM) and cisplatin (0.25, 0.5, 2.5 or 5.0 μg/mL). Results showed that the rate of apoptosis of gastric cancer cells under melatonin and cisplatin combinatorial treatment was higher than that of the control, melatonin‐alone group and cisplatin‐alone group (Figure 6). In addition, PI staining‐based flow cytometry showed that the combination of melatonin and cisplatin increased cell distribution in the G0/G1 phase (Figure 7A). IRE1α and Bip expression in the cells under combinatorial treatment was higher than that in the control (Figure 7B). In the case of autophagy, melatonin and cisplatin alone, as well as in combination, increased Beclin1 and LC3‐II expression. Furthermore, Beclin1 and LC3‐II expression in cells receiving combinatorial treatment was the highest among the groups examined. Western blotting was used to determine MAPK signalling pathway expression; the results showed that combinatorial treatment triggered higher expression of phospho‐ERK and phospho‐p38 compared to the control (Figure 7C). Taken together, these findings suggest that combining melatonin with cisplatin might be a potential strategy to enhance the effectiveness of therapy for gastric cancer.

## 4 DISCUSSION

In 2011, Douglas Hanahan and Robert A. Weinberg summarized 10 basic characteristics of tumour cells,^19^ and four new hallmarks of cancer were added by Douglas Hanahan in 2022.^20^ An important objective of chemotherapy is to overcome these characteristics of tumour cells. The characteristics of tumour growth, invasion, and metastasis are susceptible to regulation by a variety of related genes; thus, it is crucial that new targeted therapeutic agents are explored at the molecular level. In this study, we aimed to uncover the molecular mechanisms underlying the effects of melatonin on cancer metastasis to search for a more effective strategy to enhance the anti‐tumour outcome of melatonin.

Our findings showed that cell migration was inhibited by melatonin, especially at a concentration of 5.0 mM. The degradation of the basement membrane and extracellular matrix during metastatic invasion of tumours is associated with MMPs, the most closely related of which are MMP2 and MMP9.^21^ In this study, the expression of MMP‐related genes was examined, and the results showed that melatonin decreased the expression of MMP2, caldesmon 1 and tropomyosin 1, indicating that cell plasticity was changed by exposure to melatonin. In addition, EMT is defined as a cellular process in which cells lose the epithelial traits of tight cell–cell adhesion and apico‐basal polarisation, and acquire mesenchymal properties of motility and invasion.^22^ In the context of cancer, cells with activated EMT are suggested to acquire the capability to drive the invasion‐metastasis cascade.^23^ In the present study, the expression of N‐cadherin, vimentin and α‐SMA was reduced in a dose‐dependent manner in cells treated with melatonin. The expression of these EMT marker genes being downregulated in cells exposed to melatonin further confirms the inhibitory effect of melatonin on cancer cell migration. It should be pointed out that the effect of melatonin on vimentin might be pleiotropic and non‐linear. Many studies have shown that melatonin can reduce vimentin expression in cancer cells.^24^, ^25^ High expression of vimentin is observed in primary and metastatic tumours derived from epithelial tissues, and it modulates the metastatic cascade: EMT, invasion and migration.^26^ However, pretreatment with melatonin has also been shown to increase vimentin expression in porcine trophectoderm cells.^27^

That tumour cells originate from stem cells has been a long‐standing proposition. Cancer stem cells, are a small heterogeneous population within the tumour that exhibits self‐renewal and unlimited proliferative capacity and functions in cancer recurrence, metastasis and drug resistance.^1^, ^28^ Many studies have shown that melatonin can disrupt cancer stem cells.^29^, ^30^ To further evaluate the impact of melatonin treatment on cancer metastasis, CD133^+^ cells were isolated in this study. CD133 is highly expressed in various tumours and is often used as a cancer stem cell marker in stemness studies of gastric cancer.^31^, ^32^ Gene expression analysis showed that the CD133^+^ subpopulation of melatonin‐treated cells had the lowest E‐cadherin expression. In addition, autophagy induction by melatonin is weakened in the CD133^+^ subpopulation compared to the CD133^−^ subpopulation. Melatonin appears to have an unsatisfactory therapeutic outcome against gastric cancer stem cells. In fact, melatonin was found to increase c‐Myc^S62^ phosphorylation in this study. Enhancement of c‐Myc^S62^ phosphorylation is well known to promote c‐Myc stabilisation to increase tumorigenic potential.^33^, ^34^ However, there are unresolved controversies about CD133 as a marker for cancer stem cells.^35^, ^36^, ^37^ Prolonged culture of a pure CD133^−^ population can lead to the re‐emergence of CD133^+^ cells.^37^ Therefore, melatonin‐promoted ATG3 and Beclin 1 expression in CD133^−^ cells might be associated with other cancer stem cell subpopulations. This suggests that cancer treatment with melatonin alone may not be the optimal strategy.

LncRNA, a type of non‐coding regulatory RNA, is closely associated with tumour invasion and metastasis.^38^, ^39^ In previous studies, regulation of lncRNA expression together with drug treatment showed a promising combinatorial effect as a cancer therapy.^40^, ^41^, ^42^ The present study found that LINC01121, lncRNA COX2, H19, TUG1, HOTAIR, MALAT1, SAMMSON and UCA1 expression was altered after melatonin exposure. Furthermore, knockdown of H19 in melatonin‐treated cells could further enhance the expression of pro‐apoptotic genes Bax and Bak, suggesting that modulation of H19 expression may contribute to the enhancement of the inhibitory effect of melatonin on cancer progress. However, melatonin has been shown to both downregulate^43^ and upregulate H19.^44^ These contradictory observations made under different contexts highlight the pleiotropic nature of melatonin.

In the case of melatonin based‐cancer treatment, several previous reports focused on combinatorial therapy. For example, in head and neck cancer therapy, the combination of melatonin and rapamycin enhanced apoptosis via regulation of the mitochondrial function.^45^ In colon cancer, melatonin synergized the chemotherapeutic effect of 5‐fluorouracial via inhibition of the AKT and iNOS signalling pathways.^46^ Therefore, to strengthen the impact of melatonin on cancer, the synergized effect of melatonin in a combinatorial treatment was also determined. Cisplatin is a well‐known chemotherapeutic drug used for the treatment of various human cancers.^47^ As expected, the combinatorial treatment of melatonin and cisplatin could induce large‐scale apoptosis in gastric cancer cells by triggering G0/G1 phase arrest. These results suggest that co‐treatment with melatonin and cisplatin might be an appropriate strategy for cancer treatment.

## AUTHOR CONTRIBUTIONS

**Linglin Cheng:** Formal analysis (equal); investigation (equal); writing – original draft (equal). **Shubo Li:** Conceptualization (equal); formal analysis (equal). **Kailun He:** Investigation (equal). **Ye Kang:** Conceptualization (equal). **Tianye Li:** Investigation (equal). **Chunting Li:** Investigation (equal). **Yi Zhang:** Investigation (equal). **Wanlu Zhang:** Investigation (equal). **Yongye Huang:** Conceptualization (equal); formal analysis (equal); writing – review and editing (equal).

## FUNDING INFORMATION

This study was funded by the National Natural Science Foundation of China (No.81502582). Funding was also provided by the Fundamental Research Funds for the Central Universities (N182004002, N2220002), Natural Science Foundation of Liaoning Province (2021‐MS‐104, 2022‐YGJC‐39), Fundamental Scientific Research Fund of Liaoning Provincial Education Department (LJKQZ2021002), Liaoning Revitalization Talents Program, Key technologies research in major disease control and prevention (XLYC2007098), and Key Laboratory of Bioresource Research and Development of Liaoning Province (2022JH13/10200026).

## CONFLICT OF INTEREST STATEMENT

The authors confirm that there are no conflicts of interest.

## Supporting information


Figure S1.
Figure S2.
Figure S3.

## Data Availability

Data used to support the findings of this study are available from the corresponding author upon request.
