# Supplementary material for: Melatonin regulates cancer migration and stemness and enhances the anti‐tumour effect of cisplatin
Source: J Cell Mol Med. 2023 Jun 12;27(15):2215–27. doi: 10.1111/jcmm.17809 (PMC10399526; doi:10.1111/jcmm.17809)

melatonin regulates cancer migration and stemness and enhances the anti-tumor effect of cisplatin

Running Title: melatonin in cancer treatment

Linglin Cheng[1, *], Shubo Li [2, *], Kailun He[1], Ye Kang[3], Tianye Li[1], Chunting Li[1], Yi Zhang[1], Wanlu Zhang[1], Yongye Huang[1, 4, #]

[1]College of Life and Health Sciences, Northeastern University, Shenyang, 110169, China

[2]Liaoning Center for Animal Disease Control and Prevention, Liaoning Agricultural Development Service Center, Shenyang, 110164, China.

[3]Department of Pathology, Shengjing Hospital of China Medical University, No. 36, Sanhao Street, Heping District, Shenyang, 110004, China

[4] Key Laboratory of Bioresource Research and Development of Liaoning Province, College of Life and Health Sciences, Northeastern University, Shenyang, 110169, China.

* These authors contributed equally to this work

[#]To whom correspondence should be addressed: Tel: +86-24-83656116. Fax: +86-24-83656116.

E-mail: huangyongye88@163.com, huangyongye@mail.neu.edu.cn.

Figure S1. Expression of EMT associated factors as evaluated by RT-qPCR. Data are presented as the mean ± SEM of duplicate experiments. *$p<0.05$, **p <0.01 and ***p <0.001, indicate significant differences when compared with the control group.

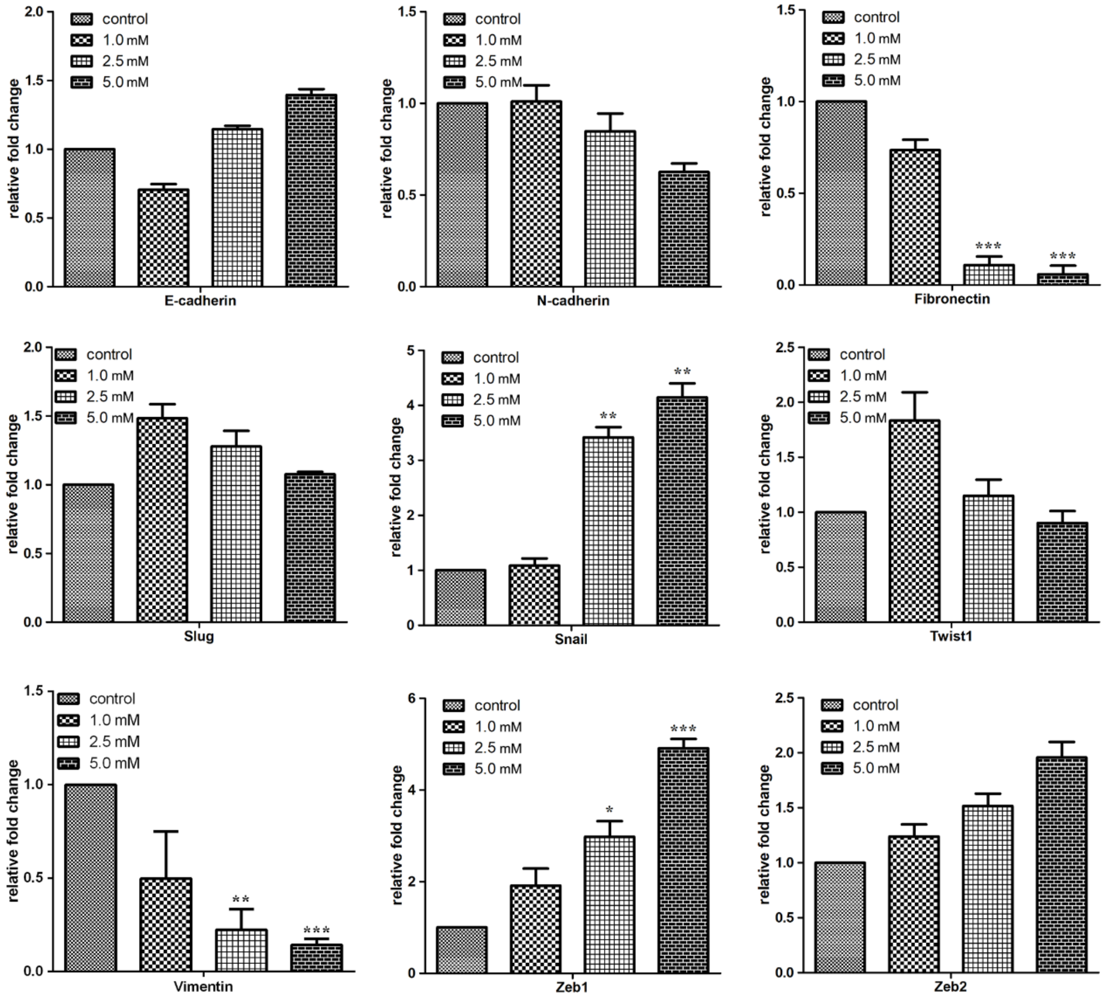

Figure S2. Expression of lncRNA as determined by RT-qPCR. Data are presented as the mean ± SEM of duplicate experiments. *p<0.05, **p <0.01 and ***p <0.001, indicate significant differences when compared with the control group.

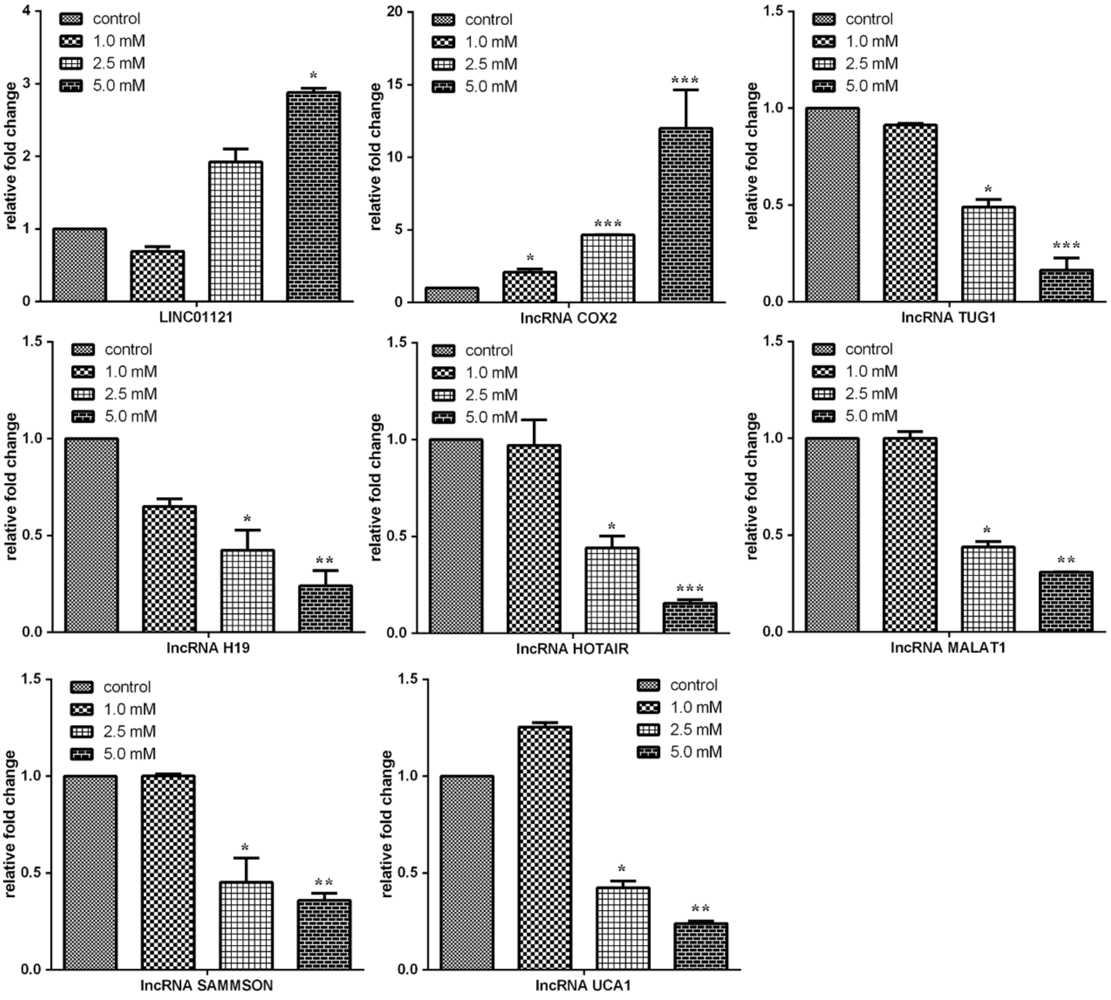

Figure S3. Expression of necroptosis and ER stress associated proteins in gastric cancer cells with lncRNA H19 knockdown and melatonin treatment.

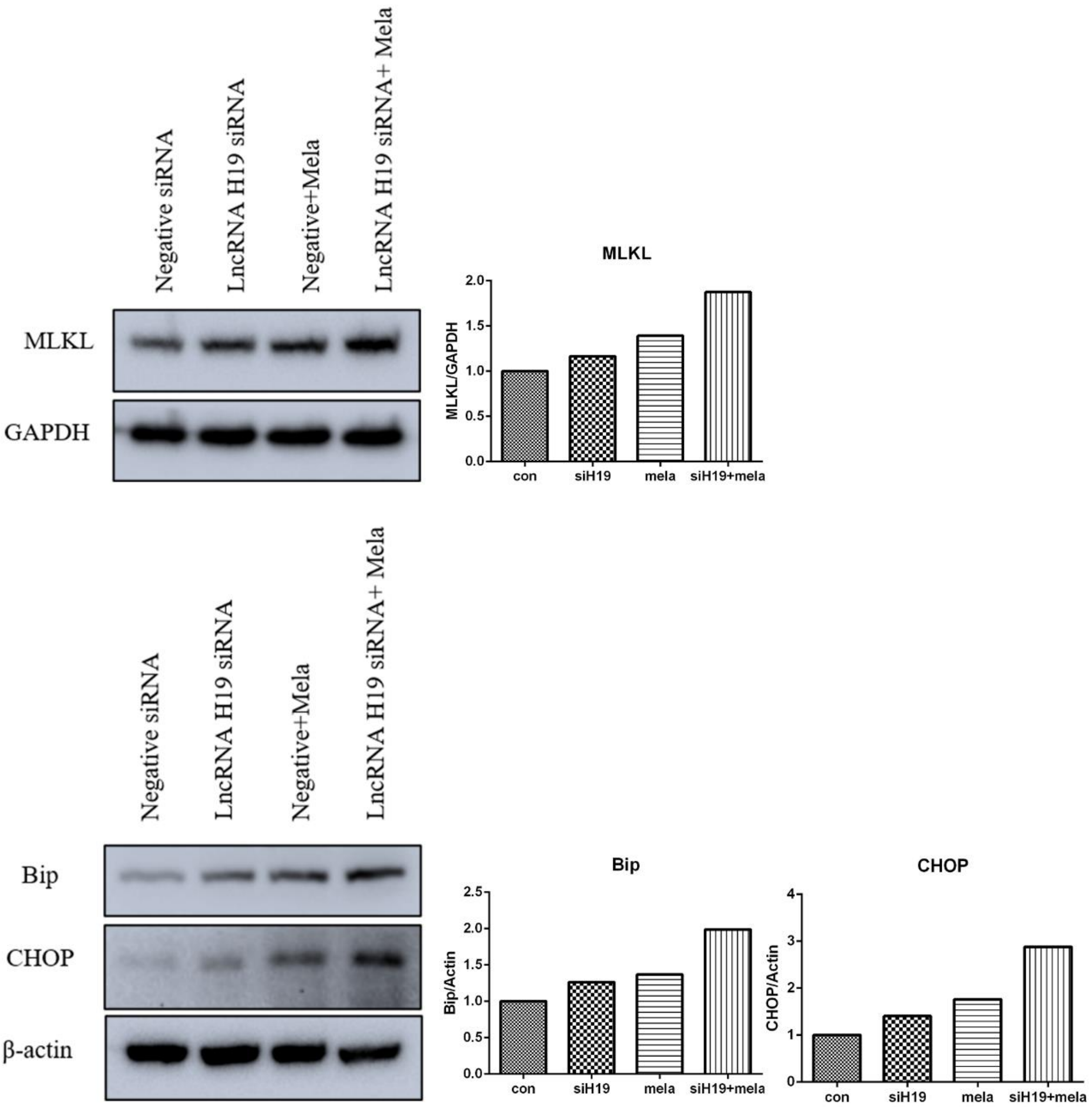

Supplement: Supplementary file 1 — Figure S1. Figure S2. Figure S3. [file JCMM-27-2215-s001.pdf]
